# Supplementary material for: Prostaglandin analog effects on cerebrospinal fluid reabsorption via nasal mucosa
Source: PLoS One. 2021 Dec 31;16(12):e0248545. doi: 10.1371/journal.pone.0248545 (PMC8719688; doi:10.1371/journal.pone.0248545)
Supplement: Attachment — Submitted filename: CSF drainage rebuttal letter PLoS ONE.docx [file pone.0248545.s006.docx]

20 September 2021

Francesco Lolli, MD, PhD

Academic Editor

PLoS ONE

Dear Dr. Lolli:

We thank you and the reviewers for your comments and suggestions to improve our manuscript. We have made the following changes and offer our responses to these comments below:

Reviewer 1

In this article, nasal delivery of prostaglandin analogues boosted nasal recovery of a CSF fluorescent tracer, implying enhanced CSF outflow via the nasal lymphatics. Latanoprost had the most substantial impact and dose-dependent. These medicines may help lower ICP.

RESPONSE: Thank you for your comments and your review.

Some section of the article needs a better presentation.

The statistic section describes "Student's t-tests and ANOVA RM with Dunnet's post hoc analysis". It is not informative and should be expanded, referring to preplanned methods.

RESPONSE: We have revised the final paragraph of the Methods section (lines 109-114 of the tracked changes version) to provide additional statistical information as requested.

The results refer to Fig.2 and Fig. 3, but it is not clear how the asterisk refers to the difference between the groups in which specific test.

RESPONSE: We have provided clarification regarding the meaning of the asterisks in the Figure legends; comparisons in each instance are between the specific drug/dose and controls unless otherwise indicated.

Fig legends refer more to methods than results.

RESPONSE: Figure legends have been rewritten to more appropriately reflect and describe the results and no longer contain methodologic information.

Fig. 1a and 1b should show the area of interest. Please indicate the signals within the ventricle and the injection track (Fig.1A) and the control ventricles with no staining (Fig.1B).

RESPONSE: Asterisks and arrows have been added to direct the reader’s attention to the fluorescent signal in the ventricle and the injection track, respectively.

Reviewer 2

The authors investigated the ability of ophthalmic prostaglandin F2alpha analogs to increase CSF outflow when vapplied to the nasal mucosa by inhalation. The idea behind this work is that no medications are available that will increase CSF outflow, and the olfactory lymphatics provide an accessible target for such a treatment. The authors utilized commercially available PGF2alpha analogues (latanoprost, bimatoprost, travoprost, latanoprostene bunod, and tafluprost) that are widely used in the treatment of open angle glaucoma.

The main results is that a single nasal inhalation of the PGF2alpha analogue latanoprost resulted in an increased recovery of a CSF tracer from nasal mucosa, that suggested an increased passage of CSF through olfactory lymphatic channels, a little effect was seen with bimatoprost and tafluprost.

RESPONSE: Thank you for your review and your very helpful comments.

The work is technically well done and well executed and data obtained are very promising. The identification of compounds with the potential to increase CSF outflow is of great interest. However, it is surprising that the authors fell short of performing additional experiments to validate their important observation. Indeed, time course experiments to pinpoint the duration of a single (or even more important) repeated dose regimen are missing.

RESPONSE: We appreciate this recommendation to look at duration of effect of dosing. As you noted, multiple dose/multiday dosing would reflect real-life use of a medication. Therefore, we undertook additional experiments with latanoprost only (given that it showed the greatest effect with single dosing) and found a sustained effect upon CSF drainage via the lymphatic pathways (new Figure 4 and text lines 164-181).

Also, in my opinion it is vital to address the effects of these prostaglandin analogs on the nasal mucosa such as morphology and cell infiltrate.

RESPONSE: The effect upon latanoprost and other prostaglandin analogues on the ocular/conjunctival mucosa has been studied extensively during pre-clinical and clinical studies. No concerning findings with respect to inflammation or cellular infiltrates were identified. These drugs are known to cause slight conjunctival hyperemia and other changes to goblet cells that are of no functional consequence. With limited research resources and a need to prioritize the most salient experiments because of COVID-19 pandemic-related restrictions on laboratory usage and capacity, we respectfully submit that the existing data on mucosal effects of prostaglandin analogues should be sufficient to allay any concerns regarding safety and tolerability when applied to the nasal mucosa, a tissue with similar properties. We have added a statement about the known effects of these substances on the mucosal surface (Lines 72-73) and included a new reference (12).

In summary, this work is a nice piece of science portraying extremely interesting preliminary results but that requires additional work to be accepted for publication

RESPONSE: We thank the reviewer again for the very helpful suggestions to improve the impact of our work.

Additional Editor Comments

The manuscript is technically sound, and the data do support the conclusions. However both reviewer had major comments, and some involve additional data requested

RESPONSE: We believe we have addressed the requests for changes and additional data that the reviewers have helpfully suggested.

Sincerely yours,

Prem S. Subramanian, MD, PhD

Professor of Ophthalmology, Neurology, and Neurosurgery

Vice Chair for Academic Affairs

Division Head, Neuro-Ophthalmology
