# Supplementary material for: Prostaglandin analog effects on cerebrospinal fluid reabsorption via nasal mucosa
Source: PLoS One. 2021 Dec 31;16(12):e0248545. doi: 10.1371/journal.pone.0248545 (PMC8719688; doi:10.1371/journal.pone.0248545)
Supplement: Attachment — Submitted filename: CSF drainage rebuttal letter PLoS ONE.docx [file pone.0248545.s007.docx]

2 November 2021

Francesco Lolli, MD, PhD

Academic Editor

PLoS ONE

Dear Dr. Lolli:

We thank you and the reviewers for your additional comments and suggestions to bring the manuscript within the PLoS ONE guidelines. We have made the following changes and offer our responses to these comments below:

Comment: Reviewing the new versions of the  manuscript entitled “Prostaglandin analog effects on cerebrospinal fluid reabsorption via nasal mucosa”  the points raised by the reviewers were considered and responded to. The manuscript is accordingly improved.

RESPONSE: Thank you very much.

In the present revision we noticed that certain details of animal experimental techniques, particularly a description of care/monitoring information, are absent or insufficiently described from the methods. They should be introduced.

RESPONSE: We have added more specific information describing the care and monitoring of the animals and trust that these details will fulfill the requirements (lines 69-74 and 91-92 in the tracked changes version). We used a recent article in PLoS ONE (<https://doi.org/10.1371/journal.pone.0247149>) as a model after reviewing overall requirements.

Thank you again for consideration of our work.

Sincerely yours,

Prem S. Subramanian, MD, PhD

Professor of Ophthalmology, Neurology, and Neurosurgery

Vice Chair for Academic Affairs

Division Head, Neuro-Ophthalmology
